# The simultaneous use of CRISPR/Cas9 to knock out the PI3Kca gene with radiation to enhance radiosensitivity and inhibit tumor growth in breast cancer

**DOI:** 10.22038/ijbms.2024.79249.17167

**Published:** 2024

**Authors:** Amir Danyaei, Rahil Ghanbarnasab-Behbahani, Ali Teimoori, Niloofar Neisi, Nahid Chegeni

**Affiliations:** 1 Department of Medical Physics, School of Medicine, Ahvaz Jundishapur University of Medical Sciences, Ahvaz, Iran; 2 Cellular and Molecular Research Center, Medical Basic Sciences Research Institute, Ahvaz Jundishapur University of Medical Sciences, Ahvaz, Iran; 3 Department of Virology, Faculty of Medicine, Hamedan University of Medical Sciences, Hamedan, Iran; 4 Department of Virology, School of Medicine, Ahvaz Jundishapur University of Medical Sciences, Ahvaz, Iran

**Keywords:** Breast carcinoma, CRISPR/Cas 9 Technique, MDA-MB-231 cell line, PI3Kca gene, Radiation

## Abstract

**Objective(s):**

Breast neoplasm is a malignancy that can have a poor prognosis. The PI3K/AKT signaling pathway is frequently activated in various tumor types, including breast cancer, leading to alterations in the tumor microenvironment and radioresistance. Selective inhibition of PI3Kca (p110α) has been considered an alternative approach to overcome radioresistance, owing to concerns surrounding the excessive side effects of pan-PI3K inhibitors tested in clinical trials. This investigation aimed to evaluate the efficacy of co-administering PI3Kca knocking out with radiation therapy in mitigating radioresistance and suppressing tumor growth in the MDA-MB-231 cell line.

**Materials and Methods:**

The present investigation utilized the CRISPR/Cas9 technique to induce a knockout of the PI3Kca gene. Subsequently, after 24 hr of transfection, gene expression, cell proliferation, apoptosis rate, and angiogenesis were assessed.

**Results:**

We demonstrated that knocking out PI3Kca, in combination with radiation, increased apoptosis, reduced the expression of PI3Kca and AKT1 genes, and decreased cell proliferation. The CAM assay analysis has demonstrated that knocking out the PI3Kca gene and radiotherapy substantially reduced the total vessel network length and the number of junctions.

**Conclusion:**

The findings of our investigation indicate that the integration of radiation therapy with PI3Kca yielded enhanced radiosensitivity, leading to a marked retardation of tumor progression and an increased survival rate.

## Introduction

Even though cancer treatments and diagnostic techniques have recently progressed, most cancers remain incurable diseases with significant death rates in many regions worldwide. Among the many breast neoplasm variants, triple-negative breast cancer (TNBC) is characterized by a lack of expressiveness in the estrogen receptor (ER), progesterone receptor (PR), and human epidermal growth factor receptor 2 (HER2) (1). TNBC occurs in approximately 10–15 percent of all breast cancers and is identified by aggressive behavior, a tendency for early recurrence, metastasis, and poor prognosis. Cancer cell heterogeneity has been identified as a factor in different clinical outcomes and therapeutic responses in TNBC tumors (2). For breast cancer patients, surgery combined with chemo-radiotherapy is still the most effective treatment strategy (3). 

Much effort has been spent on finding novel biomarkers and related medicines in recent years, but only a few have proven beneficial in clinical studies. However, research is ongoing to find genes or signaling pathways affecting TNBC. Phosphoinositide 3-kinases (PI3Ks) are enzymes that play an essential role in cellular function, such as cell growth, differentiation, proliferation, motility, and survival. PI3K mutations are observed in over one-third of breast tumors, as well as cancers such as colon, cervical, glioma, stomach, liver, lung, and prostate (4, 5). PI3K has a subunit called PI3Kca, the most recurrently mutated gene in breast cancer. Although the PI3Kca rate escalates in breast cancer subgroups, HR+/HER2– (42%) and HER2+ (31%), it demonstrates a lower mutation in the TNBCs group (16%) (6, 7). Moreover, prior studies have indicated the effect of PI3Kca mutation in HR+ and HER2+, while it is still poorly recognized in TNBC. Furthermore, the PI3K/AKT/mTOR signaling pathway plays a significant role in the aggressive behavior of tumors like angiogenesis, invasion, migration, and metastasis. This pathway is activated by PI3Kca mutation due to induced hyperactivation of the alpha isoform (p110alpha) of PI3K (8, 9). Determining patients’ survival with TNBC with or without PI3Kca mutation is arguable because of the distinct outcomes of restricted research. In addition, some studies have illustrated that PI3Kca mutation was associated with a reduced pathological complete response rate in TNBC treatment with neoadjuvant chemotherapy; hence, this mutation can be related to radio/chemotherapy resistance in TNBC (10-12).

Since several studies have associated enhanced PI3Kca mutant expression with radioresistance in TNBC cells (e.g., MDA-MB-231) (13), we knocked out PI3Kca in the MDA-MB-231 cell line using the CRISPR/Cas9 approach this time. We also examined the synergistic effects of irradiation and gene knockout on tumor cell behavior and alteration in related molecular signals.

## Materials and Methods

In this study, the experimental groups were exposed to non-irradiated and irradiated conditions, as indicated in Table 1. All procedures were performed 24 hr after irradiation and 48 hr after transfection for the non-irradiated groups. 

***Cell culture***

The MDA-MB-231 cell line was acquired from the Iranian Biological Resource Center (IBRC-Iran). The cells were cultured in DMEM supplemented with 1% antibiotic (BIO-IDEA-Iran) and 10% fetal bovine serum (BIO-IDEA-Iran). They were incubated at 37 °C in an environment with 5% carbon dioxide and 95% humidity.

***Construction of sgRNA/Cas9 vectors***

The sgRNA sequence has been designed using an online sgRNA design tool at https://crispr.mit.edu/ to PI3Kca knock out. The series of gatcgCGAACAGGTATCTACCATGGg (forward) and aaaacCCATGGTAGATACCTGTTCGc (reverse) has been synthesized to insert into the GE100018 vector containing the U6 promoter to progress the sgRNA transcription. According to a previous study, the annealing primers were purified and cloned (13). Subsequently, colony PCR and the Sanger sequencing method (Macrogen, South Korea) were used to confirm constructed recombinant plasmids. The primer was doubled based on under 90 ℃ for 1 min, 80, 70, 60, 50, and 40 ℃ for 10 sec. In order to maximize the production of the Cas9 protein, the gRNA sequence was chosen in a way that resulted in the greatest on-target activity scores, the lowest off-target impacts, and the most mismatches with other genes. 

***Transfection of MDA-MB-231 cells***

After the confluence of MDA-MB-231 cells in the 24-well culture plate reached 60–70%, the transfection was done by TurboFect reagent (Thermo Fisher Scientific, USA) according to the manufacturer’s instructions; the transfection effectiveness was monitored using a fluorescent microscope. The transfection was optimized with 1 µl of DNA to 3 µl reagents for each well. This value was equal to 0.5 µg of DNA.

***Irradiation***

Cell irradiation was performed using the 6MV beam of a linear accelerator (Varian, Golestan General Hospital, Ahvaz, Iran). We applied one dose of 2 Gy and positioned the source-to-surface distance (SSD) at 100 cm 24 hr after transfection.

***Quantitative reverse transcription-polymerase chain reaction***

RNX-Plus reagent was applied to extract total RNA from the MDA-MB-231 cells (SinaClon- Iran). Afterward, mRNA was transcribed to complementary DNA (cDNA) (Qiagen, Germany). Polymerase chain reaction (PCR) was carried out using a qTOWER Real-time PCR System (Analytikjena, Germany) following the manufacturer’s protocol (RealQ Plus 2x Master Mix Green, Denmark). The RT-qPCR primer sequences utilized are as follows: 5´-AGCCACACACTACATCAGTGG-3´(forward) and 5´-ATGAAACAGTTGTCCATCGTCT-3´ (reverse) for PI3Kca, 5´-GGCAAGGTGATCCTGGTGAA-3´(forward) and 5´-TGGCCACGATGACTTCCTTC-3´ (reverse) for AKT1, and 5´-TAGCCCTCTGTGTGCTCAAG-3´(forward), and 5´-ACTTTTATGTCCCCTGTTGACTG-3´(reverse) for HPRT as an internal control.

***Proliferation assay***

The MTT (3-(4, 5-methylthiazol-2-yl)-2, 5-diphenyl-tetrazolium bromide) assay was evaluated to measure cellular metabolic activity as an indicator of cell viability. Briefly, the 15×10^3^ cells were seeded per well; 24 hr after the cells were transfected, they were treated with 10 µl of MTT reagent (Sigma-Aldrich, Shanghai, China) for 3 hr at 37 °C in the dark at 0.5 mg/ml concentration. Finally, the cell growth medium was entirely removed, and 100 µl of dimethyl sulfoxide was added and mixed for 8 min. The microplate reader was employed to determine the optimal wavelength for absorbance at 570 nm (BioTek, USA, ELx808 Absorbance Microplate Reader).

***Observation of morphological features***

Several studies have shown that the cytoskeleton’s arrangement, the degree to which cells adhere to one another, and the substrate determine the cell’s form (14). Moreover, investigations indicated that PIK3ca mutations were highly linked with morphology, race, ER status, PR status, and HER2 status in breast cancer. In addition, it collaborates with Bcl-2 to cell morphology and migration (15, 16). Hence, 30,000 cells were placed into a 12-well plate. After incubation in standard conditions, transfection, and irradiation, we analyzed the cellular morphology using an inverted microscope, a method commonly used to study changes in cell morphology, to examine the differences between the irradiated and non-irradiated groups.

***Colony formation assay***

Using the clonogenic survival experiment, we investigated a single cell’s potential for growth after PI3Kca knockout. A summary of the procedure involves irradiating the cells with single doses ranging from 0 to 6 Gy after PI3Kca was knocked out. After irradiation, two milliliters of media containing 10% FBS were added to 6-well plates with the correct number of cells after trypsinization. For 9–14 days, the cells were maintained at 37 °C in an incubator with 5% CO_2_. Pasteurized saline (PBS) was used to wash the colonies, followed by 4% formaldehyde for fixation and 0.5% crystal violet staining. Based on prior studies, the plating efficiency (PE), survival fraction (SF), and sensitization enhancement ratios (SER) were determined for every group (17).

***Apoptosis assay***

The assessment of apoptosis induction was performed by employing Annexin V-FITC and propidium iodide (PI) staining (eBioscience, USA) for both the irradiated and non-irradiated groups. In summary, five μl of Annexin V-FITC and five μl of PI were added to 500,000 cells suspended in 500 μl of binding buffer. The solution was allowed to incubate for 15 min at normal temperature in a light-excluded setting. Ultimately, the data were scrutinized utilizing a flow cytometer (BD FACSCalibur- USA). The data were processed using the FlowJo software program.

***Chick chorioallantoic membrane assay (CAM Assay)***

The CAM test is a well-known model for studying cancer cell invasion and metastasis. There are many benefits to using the CAM method. This *in vivo* model is easy to replicate, inexpensive, and allows large-scale screening. Our study aimed to investigate the impact of PI3Kca knockout combined with irradiation on MDA-MB-231 cells. We applied the method described in our previous study (17).

***Statistical analysis***

The data were examined using GraphPad Prism 8. For statistical significance analysis, a two-way analysis of variance (ANOVA) or Student’s t-test was used. A *P*-value less than 0.05 proved statistically significant, and the findings were shown as the mean plus or minus the standard deviation.

**Table 1:** These are the experimental groups that were used in this investigation

**Experimental groups **	**Description**
**NC **	Did not receive vector
**Scramble (SCR)**	Received vector without sgRNA
Ko-PI3	The PI3Kca gene was knocked out using the CRISPR/Cas9 technique

NC: Negative control; SCR (Scramble); and Ko-PI3 (PI3Kca gene knock-out)

**Figure 1:**
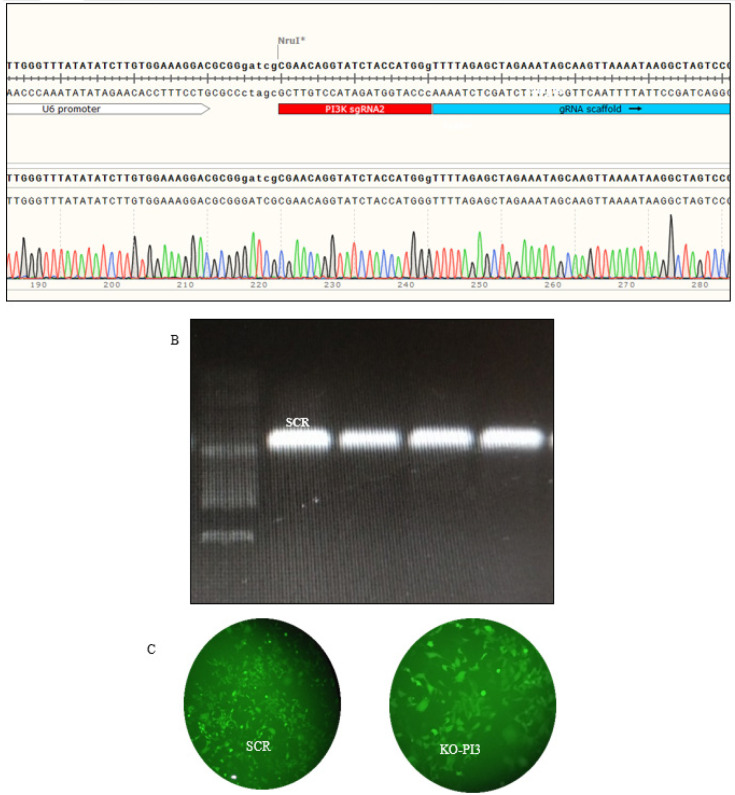
(A) Verification of the cloning of the PI3Kca gene into the plasmid. (B) The agarose gel electrophoresis was utilized to analyze the colony PCR products. (C) The MDA-MB-231 cells were transfected with GE100018 and the gRNA

NC: Negative control; SCR: Scramble; and Ko-PI3 (PI3Kca gene knock-out)

**Figure 2:**
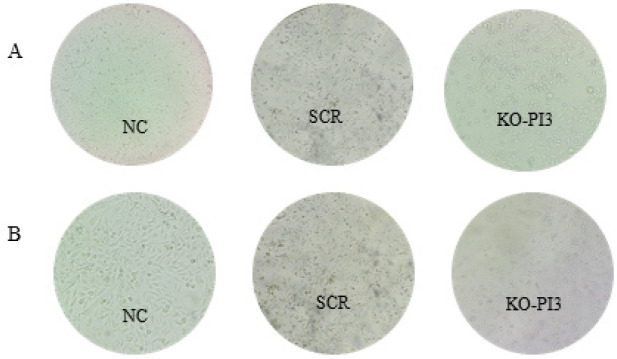
Consequences of PI3Kca gene deletion and radiation on cell morphology. Microscopic study shows changes in cell morphology 24 hr after the second intervention (irradiation)

(A) The group without radiation was the group in which only the PI3Kca gene was knocked out. (B) the group received 2G of radiation 24 hours after the PI3Kca gene was knocked out; NC: (Negative control), SCR (Scramble), and Ko-PI3 (PI3Kca gene knock-out).

**Figure 3:**
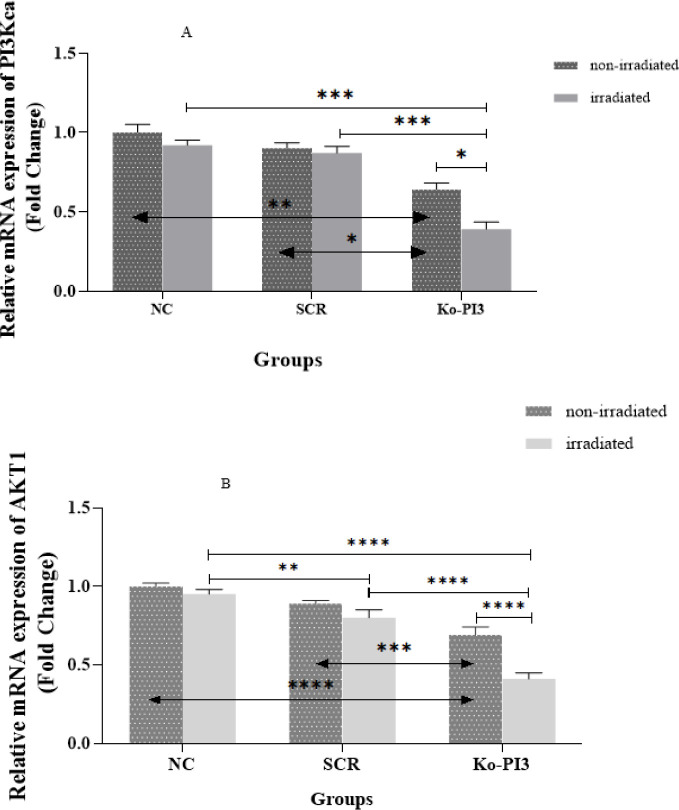
Effect of the PI3Kca knocked out in combination with radiotherapy on mRNA expression of PI3Kca gene (A) and AKT1 (B), based on the RT-qPCR results in either non-irradiated or irradiated groups in each of their three subgroups (*P<0.05; ** *P*<0.01; *** *P*<0.01; and ***P<0.001).

NC: Negative control; SCR: Scramble; and Ko-PI3: PI3Kca gene knock-out

**Figure 4:**
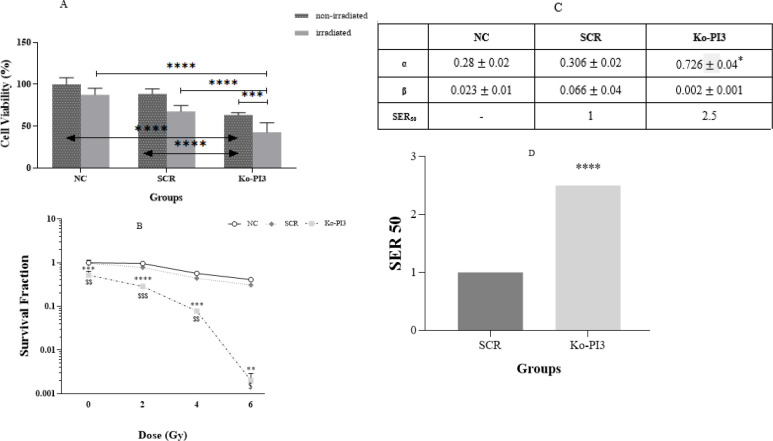
(A) percentage of cell survival in different groups (with and without radiation) shows a decrease in cell survival rate after knocking out the PI3Kca gene. (B) The fraction of cell survival after knocking out the PI3Kca gene has been shown in different doses of radiation. (C) The sensitivity enhancement ratio (SER) was measured for the PI3Kca group compared to the SCR group based on standard mathematical relationships. (D) Alpha and beta coefficients, which are criteria for checking cell survival and colonization, were measured using standard methods for each group in the experiment

The significance levels of the comparison of the groups were expressed as follows. ****P<0.0001, ***P<0.001, **P<0.01, $$$ *P*<0.001 and $$ *P*<0.01, and $ *P*<0.05).

NC: Negative control; SCR: Scramble, and Ko-PI3: PI3Kca gene knock-out

**Figure 5:**
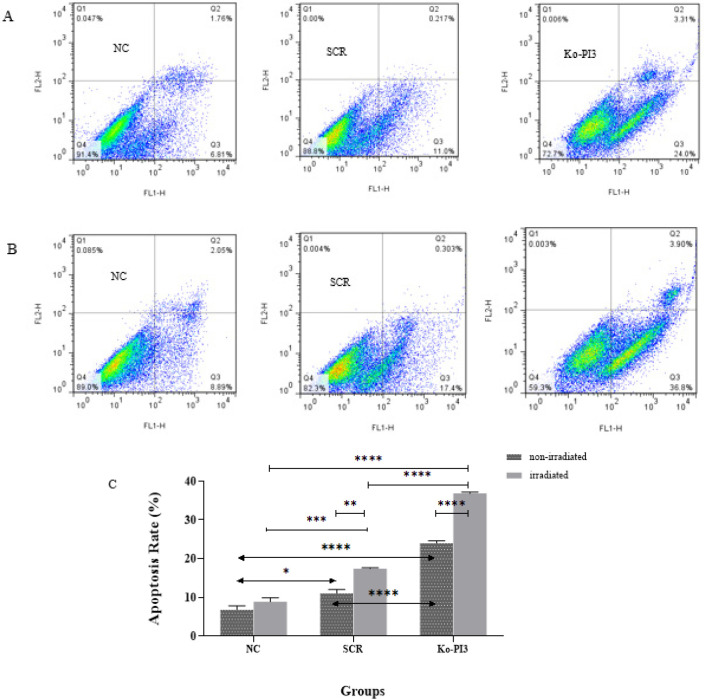
Flow cytometric analysis, a precise and reliable method, was used to assess apoptosis using Annexin V/PI staining and FlowJo software in the MDA-MB-231 cells (A) Representative images of apoptosis in non-irradiated and (B) irradiated groups. This analysis shows the percentage of cells that have undergone apoptotic death and necrosis. (C) The graph illustrates the levels of apoptosis in the experimental groups under both non-irradiated and irradiated conditions. The comparison of these levels, which is of significant importance, underscores the synergistic effects of irradiation and PI3Kca gene knockout on apoptosis (*P<0.05; ** *P*<0.01; ***P<0.001; and **** *P*<0.0001); NC: Negative control; SCR: Scramble, and Ko-PI3: PI3Kca gene knock-out

**Figure 6:**
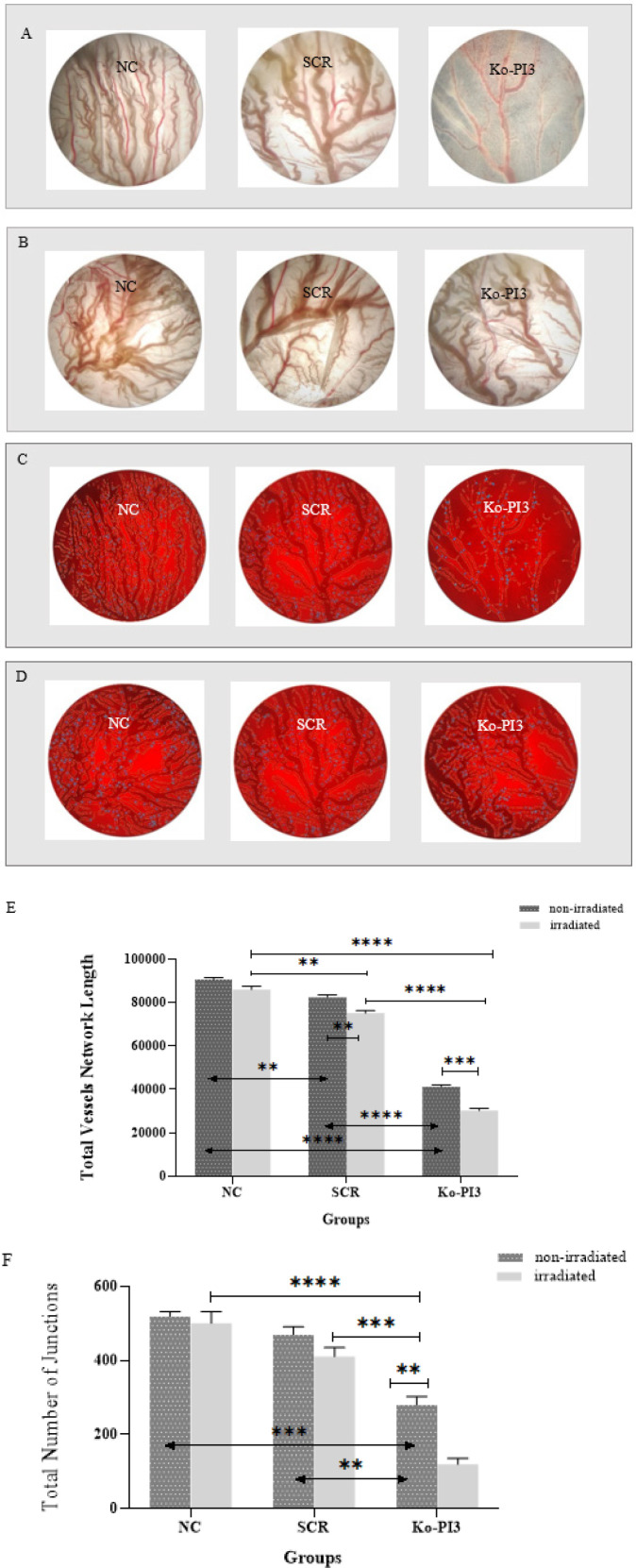
Inhibition of angiogenesis in the CAM model was observed through the combination of radiotherapy and the knocking out of the PI3Kca gene

The microscopic examination revealed alterations in the process of angiogenesis. (A) non-irradiated groups, (B) irradiated groups, (C) analysis non-irradiated groups, and (D) analysis irradiated groups. (E) The present study aims to quantify the total length of vessel networks and (F) the total number of junctions using quantitative methods (*P<0.05, **P<0.01, ***P<0.001, and ****P<0.0001).

NC: Negative control; SCR: Scramble; and Ko-PI3: PI3Kca gene knock-out

## Results

***gRNA cloning and assessment of the transfection effectiveness***

The length of the band containing the PI3Kca gRNA, as determined by electrophoresis after purification of the PCR products, was determined to be 450 base pairs (bp). Furthermore, Sanger sequencing verified that the intended gRNAs were located in the correct position on the vector, close to the U6 promoter. After 24 hr, the transfection effectiveness was assessed using an inverted fluorescence microscope. Approximately 50% transfection efficiency was calculated for MDA-MB-231 cells. The transfection efficiency was examined 24 hr later using an inverted fluorescence microscope. The MDA-MB-231 cells have been demonstrated to have an acceptable transfection rate (Figure 1). 

***PI3Kca consequences knockout accompaniment with irradiation on cellular morphology***

In order to assess the impact of PI3Kca knockout, either alone or in combination with irradiation, on cell morphology, we observed all the treated groups using an inverted microscope (Hund, Wetzlar, Germany). The SCR group had evident alterations in cell-cell interactions compared to the NC group. The cellular communication pattern, morphology, dimensions, and organization have changed in the PI3Kca group compared to the NC and SCR groups. The irradiated NC and SCR groups exhibited no significant changes compared to the preceding condition. The wells containing PI3Kca exhibited a significant presence of non-viable cells and suspended particulate matter, as shown in Figure 2. 

***Impact of PI3Kca knockout and radiotherapy on gene expression***

We evaluated PI3Kca mRNA levels in the MDA-MB-231 cell line after the gene was knocked out and irradiated by RT-qPCR. In the non-irradiated groups, PI3Kca gene expression is considerably reduced in the Ko-PI3 group compared to the NC group (*P*<0.01). Moreover, the expression level of the PI3Kca gene showed notable variations in the Ko-PI3 group compared to the SCR group (*P*<0.05). Furthermore, when comparing the KO-PI3 irradiated group to the Ko-PI3 non-irradiated group, the outcomes demonstrated a substantial down-regulation in the irradiated group (*P*<0.05). Also, it is worth noting that differences were insignificant in the NC and SCR groups in irradiated and non-irradiated conditions. However, the PI3Kca expression meaningfully declined in the Ko-PI3 irradiated group compared with the NC and SCR irradiated groups (*P*<0.001 in both). Our findings underscore the significant decrease in AKT1 expression after the knockout of the PI3Kca gene, compared to the NC and SCR groups (*P*<0.0001 and *P*<0.001, respectively). Following irradiation, the SCR and Ko-PI3 groups exhibited a significant reduction in AKT1 expression compared to the NC group (*P*<0.01 and *P*<0.0001, respectively). The rate of AKT1 down-regulation was also lower in the Ko-PI3 group compared to the SCR group (*P*<0.0001). Importantly, in the group exposed to Ko-PI3 irradiation, we observed a significant decrease in AKT1 production compared to the Ko-PI3 group (*P*<0.0001) (Figure 3).

***Cell proliferation promoted consequent to PI3Kca gene knockout accompanied with ionizing radiation***

We initially used CRISPR/Cas9 to knock out the PI3Kca gene in MDA-MB-231 cells to observe how this might impact cell proliferation. The results indicated that the Ko-PI3 group had a substantially reduced cell viability rate compared with the NC and SCR groups (*P*<0.0001 in both). Furthermore, we treated the PI3Kca knock-out with irradiation to examine how it affected the cells’ susceptibility to radiation. We noticed cell viability was considerably smaller in the Ko-PI3 irradiated group compared to the non-irradiated same group (*P*<0.001). At the same time, there were no meaningful changes between the irradiated NC and SCR groups compared with the non-irradiated same groups, while after irradiation, cell proliferation declined in the SCR group compared with the NC group (*P*<0.01). Moreover, comparing Ko-PI3 and NC groups, the SCR group indicated a meaningful reduction in cell proliferation (*P*<0.0001 in both) (Figure 4A). 

***Combined influence of radiotherapy and PI3Kca gene knockout on survival fraction reduction***

Following the PI3Kca knockout, the clonogenic survival experiment was conducted on MDA-MB-231 cells in all treatment groups (Figure 4 B). Our data showed that the combination of PI3Kca knock-out and radiotherapy significantly reduced the surviving fraction in the Ko-PI3 group compared to the NC and SCR groups at all doses (***P<0.001, ****P<0.0001, ***P<0.001, **P<0.01, $$ *P*<0.001, $$$ *P* <0.001, $$ *P*<0.01, and $ *P*<0.05, based on the group and dose received). In order to evaluate these effects more thoroughly, SER was quantified for each group (Figure 4C). According to Figure 3C, the quantity of SER for the Ko-PI3 group was 2.5, indicating reduced radioresistance in MDA-MB-231 cells. Furthermore, the combination of CRISPR/Cas9 and radiation therapy resulted in a considerable rise in the α parameter within the treatment group, as seen in Figure 4D.

***Apoptosis rate reduction after radiation and PI3Kca gene knockout***

Flow cytometry is employed to investigate apoptosis induction, a characteristic of radiosensitivity. The non-irradiated Ko-PI3 group revealed a substantial increase in apoptosis compared with the NC and SCR groups (*P*<0.0001 in both groups). Also, comparing the SCR group with the NC group showed a significantly higher apoptosis rate (*P*<0.05). After using a 2 Gy irradiation dose, apoptosis was evaluated between the non-irradiated and irradiated groups; the Ko-PI3 group indicated substantially increased apoptosis (*P*<0.0001). In addition, the irradiated SCR group illustrated a significant increase in apoptosis rate compared to the non-irradiated SCR group and the irradiated NC group (*P*<0.01 and *P*<0.001, respectively). Comparing the Ko-PI3 with NC and SCR groups indicated a meaningfully increased apoptosis rate after applying a 2 Gy dose (*P*<0.0001 in both) (Figure 5).

***Knockout of the PI3Kca gene with radiation in the CAM model declined angiogenesis***

We employed the CAM assay to analyze the impact of PI3Kca gene knockout alone and in combination with radiation on angiogenic ability. We noticed statistically significant differences between the NC and SCR groups (*P*<0.01 for total vessel length), which was to be anticipated. Furthermore, in the Ko-PI3 group, the rate of angiogenesis was dramatically reduced compared with the NC and SCR groups (*P*<0.0001 for total vessel length (in both) and *P*<0.001 and *P*<0.01 for the total number of junctions, respectively). The post-irradiation comparison of the SCR and the NC groups illustrated a considerable decrease in the total junctions (*P*<0.01). In addition, the Ko-PI3 group demonstrated a more significant reduction in angiogenesis percentage compared to the NC and SCR groups (*P*<0.0001 for total vessel length (in both); *P*<0.0001 and *P*<0.001 for the total number of junctions, respectively). Moreover, according to the study, there was a noteworthy reduction in the length of vessels and the number of junctions in the SCR and Ko-PI3 groups compared to the same non-irradiated groups (*P*<0.01 and *P*<0.001 for total vessel length, respectively, and *P*<0.01 for the total number of junctions (for Ko-PI3)) (Figure 6).

## Discussion

The present investigation applied the CRISPR/Cas9 methodology to knock out the PI3Kca gene in the MDA-MB-231 breast cancer cell line, followed by exposure to a conventional 2 Gy radiation dose. Our findings displayed that gene knockout exerted a remarkable inhibitory effect; besides, radiation exposure after knocking out the PI3Kca gene more meaningfully delayed invasive tendencies in tumor cells.

The PI3Kca gene has been identified as the second most frequently mutated gene after P53 in breast cancer (18). PI3Kca mutation in hormone receptors and breast cancer has been identified as a causative factor for the subsequent modification of cyclin D1 and Rb protein (19). Some research suggests that a negative response to chemotherapy may be linked to PI3Kca gene mutations; however, the precise molecular mechanism underlying this relationship remains unclear. Furthermore, several studies have demonstrated that PI3Kca mutation resulted in the persistent activation of the PI3K/AKT/mTOR signaling pathway and inhibition of programmed cell death, thereby facilitating the development of resistance to chemotherapy in triple-negative breast cancer (20). Our data illustrated that the PI3Kca knocking out via CRISPR/Cas 9 technique caused a meaningful decline in mRNA expression. After that, we applied 2 Gy of conventional dose, which remarkably reduced the PI3Kca mRNA expression; likewise, the inhibition of PI3Kca substantially affected the down-regulation of AKT1 expression. 

The AKT1 gene is classified as an oncogene and plays a crucial function in numerous signaling pathways. It assists in controlling cell survival, proliferation, and differentiation—the process by which cells develop into mature forms capable of performing particular tasks (21). This procedure is observed in other methods; in other words, MTT, colony formation, and CAM assays indicated a substantial decline in cell proliferation, survival fraction, and angiogenesis in the Ko-PI3 group in which the PI3Kca gene was knocked out. The rise in α value indicates that the combination of PI3Kca knocking out and radiotherapy may cause much damage to cells, resulting in a higher rate of cell death and increased susceptibility to radiation. Besides, radiation after knocking out genes decreases aggressive behavior in MDA-MB-231 cells. In contrast, the apoptosis rate rose in the Ko-PI3 group, which was further elevated when radiation treatment was administered. Jeremy Johnson and his colleagues assessed the impact of knocking down AKT isoforms, combined with PI3K and AMPK subunits, along with irradiation in a study published in 2020. The researchers employed siRNA to induce the knockdown of specific genes. They utilized irradiation to examine tumors’ aggressive behaviors, focusing on apoptotic alterations after knocking down genes. The study indicated that knocking down AKT1 or p110α, encoded by PI3Kca, reduced cyclin D1 expression and increased PARP expression in TNBC cells, such as MDA-MB-231 (19). Due to the role of cyclin D1 overexpression and PARP down expression in the sustenance of cancerous cells and their relationship with AKT 1 and p110α, inhibiting AKT 1 and p110α leads to augmented radiosensitivity in triple-negative breast cancer cells (20, 22). Nevertheless, evaluating the knockdown of AKT1 and p110α revealed that AKT1 exhibits a greater apoptosis rate than p110α. Therefore, AKT1 was selected as the principal target in conjunction with radiation. The study examined the impact of the PI3K/AKT pathway on radiosensitivity in TNBC cells, both before and after radiation. The results indicated that knocking down AKT1 isoforms and PI3K and AMPK subunits, either alone or in combination, increased radiosensitivity (19). This study was most closely related to our research, and the data of both articles were completely aligned despite the differences in some objectives. Mi Youn Seol and her colleagues have demonstrated that co-administration of PI3K isoform-selective inhibitors (p110α, p110β, p110γ, and p110δ) with radiation therapy can effectively decrease radioresistance and inhibit tumor growth in patients with non-small cell lung cancer (NSCLC). A substantial enhancement of radiosensitivity was observed upon inhibition of the PI3K/AKT pathway. The PI3K-α inhibitor exhibited a similar radiosensitizing effect to the PI3K pan-inhibitor, both *in vitro *and* in vivo.*

Furthermore, a notable augmentation in DNA double-strand breaks (DSB) and a reduction in migration capacity were observed. The findings of this investigation indicate that the co-administration of radiation and the PI3K-α isoform led to an enhancement in radiosensitivity, thereby causing a noteworthy postponement in the progression of tumors and an amelioration in the survival rate (23). In a completely similar study by these researchers, inhibiting PI3K isoforms via CRISPR/Cas9 in mice with glioblastoma has confirmed that the inhibition of the PI3K-α isoform leads to an enhancement of radiosensitivity, thereby causing a reduction in tumor growth and an extension of survival (24). In a study, it was shown that the drug resistance of human epidermoid carcinoma and NSCLC MDR cells was overcome by suppressing the activation of the PI3K 110α and 110β catalytic subunits using BAY-1082439 and CRISPR/Cas9 deletion of PI3Kca and PI3Kcb. It resulted in the down-regulation of ATP-binding cassette transporters P-gp/ABCB1 and BCRP/ABCG2, thereby restoring drug sensitivity. The reversal of multidrug resistance (MDR) mediated by P-glycoprotein (P-gp) or breast cancer resistance protein (BCRP) was not observed upon inhibition of AKT. The ABC family proteins and AKT have the potential to facilitate the independent enhancement of MDR (25). In addition, it was found that in lung adenocarcinoma A549 cells, knocking out the INPP4B protein, which is involved in the PI3K-Akt-mTOR signaling pathway, utilizing CRISPR/Cas9 gene editing makes the cells more sensitive to ionizing radiation (IR), the PARP inhibitor olaparib and DNA homologous recombination repair is impaired. The enhanced IR sensitivity seems related to the loss of INPP4B protein since the INPP4B knockout cells’ resistance to IR was partly restored when a CRISPR/Cas9-resistant INPP4B gene was reintroduced (26). In a rigorous study, Huayu Hu and colleagues meticulously investigated the effect of the PI3Kca gene on the growth and resistance of TNBC cells (MDA-MB-231 and MDA-MB-486) and animal and human data. Despite the differences in our study methods, we arrived at the same robust results. Our findings confirm that PIK3CA mutation promotes TNBC cell growth and migration. Importantly, cells with PIK3CA mutations exhibited reduced apoptosis, indicating a robust resistance to chemotherapy, particularly with epirubicin. Xenograft tumor experiments in mice further validated this resistance. Their study also thoroughly examined apoptosis markers (Xiap, Bcl-2, and Caspase 3) and proteins associated with the PI3K/AKT/mTOR pathway (p110α, AKT, p-AKT, mTOR, p-mTOR, p-4E-BP1, p-p70S6K, and Pten) using western blot and immunohistochemistry, providing detailed and reliable insights. Furthermore, a prognostic analysis of 50 TNBC patients hinted at a potential correlation between PIK3CA mutation and a higher risk of relapse and mortality, further instilling confidence in the validity of our research (27). In general, it can be claimed that the knockout of essential genes, such as PI3Kca, can result in significant cellular damage, as observed in the notably chemoradioresistant MDA-MB-231 breast cancer cell line. As a result, other therapeutic modalities, such as radiotherapy, may augment the process of cellular death. 

Nonetheless, it should be noted that using the CRISPR technique also brings limitations and possible problems that must be seriously considered in its clinical use. Although the amount of Off-Target effects and mismatch is one of the necessary things and is highly regarded in the design, the specific nature of delivery challenges, such as the difficulty in delivering the CRISPR components to the target cells, immune response and incomplete editing. Mosaicism is another crucial issue that demands attention, particularly in *ex vivo* conditions. These factors can diminish the therapeutic potential of CRISPR-Cas9 and cast doubt on scientific findings about gene activities. However, when we consider the extraordinary capabilities of CRISPR and the paradigm shift it has brought about in genetic research, its potential can be harnessed if we gain a thorough understanding and address these limitations.

## Conclusion

Knocking out the PI3Kca gene significantly inhibits cell division, expansion, and resistance to programmed cell death in MDA-MB-231 cells. However, combining the PI3Kca knocking out with a 2 Gy dose is even more effective. Furthermore, these findings indicate that PI3Kca plays a crucial role in the expression of the AKT1 gene and the growth of MDA-MB-231 cells.

## Data Availability

The datasets utilized and/or examined in the present investigation can be obtained from the corresponding author upon reasonable request.

## Declarations

The experimental protocols were approved by the Human Ethics Committee of Ahvaz Jundishapur University of Medical Sciences (IR.AJUMS.REC.1397.183). They were conducted according to the ethics guidelines of the national health and research institutes.
